# Supplementary material for: Chromothripsis during telomere crisis is independent of NHEJ, and consistent with a replicative origin
Source: Genome Res. 2019 May;29(5):737–49. doi: 10.1101/gr.240705.118 (PMC6499312; doi:10.1101/gr.240705.118)
Supplement: Supplemental Material [file supp_gr.240705.118_Supplemental_file_1.zip › contigs/annotated_contigs/DB40/contig.4.DB40_length_791_mean_cov_6.32237673831.docx]

**DB40_length_791_mean_cov_6.32237673831**

```
TTTTTTTCTTTTTTTTTTTTTTTTTTTTTTTTTTTCTTCTTTTTTTTTTTTTTTTTTTTTTTTTTTTTTTTTGG|GTAAGTGTTTTTTG
 >chr6:160521767-160521841 + E=1e-23 p=0e+00
CTGCAAACTTTCCTT|TTTTTTTTTTTTTTGAGACGGAGTCTTGCTCTATTGCCCAGGCTGGAGTGCAGTGGGTGCGATCTCGGCTCAC
                >chr7:4640637-4640998 - E=2e-194
TGCAAGCTCCACCTCCCGGGTTCACGCCATTCTCCTGCGTCAGCCTCCCGAGTAGCTGGGAGTACAGGCGCCCGCCATCACGCCCGGCT

AATTTTTTGTATTTTTAGTAGAGACGGGGTTTCACCGTGTTAGCCTGGATGGTCTTGATCTCCTGACCTCATAATCGGCCTGCCTTGGC

CTCCCAAAGTGTTGAGATTACAGGCGTGAGCCACCGTGCCCGGTCCTGAGTTGTTTCTTAAAAAAATTTTAGATTCAGGGCCAGGGGCA

GTGGCTCACGCCTGTAATCC|GGCTGACCCCCCCACCTCCCTCCCAGACGGGGCGGCTGGCCGGGTGGGGGGCTGACGCCCCCACCTCC
                      >chr4:175613275-175613479 - E=4e-68 p=0e+00
CTCCCAGATGGGGCGGCTGGCCTGGCGGGAGGCTGACACCCCCCACCTCCCTCCCGGACGGGGTGGCTGCCGGGCGGAGAGGCTCCTCA

CTTCTCAGACGGGGCGGCTGCTGGGCGGAGGGTCTCCTCACTTCTCAGACG|AGGC|GGCCGGGCAGAGATGCTCCTCACCTCCCAGAC
                                                        >chrX:125775077-125775193 - E=6
GGGGTGGCGGCCGGGCGGAGGCTGCAATCTCGGCACTTCGGGAGGCCAAGGCAGGCGGCTGGGAGGTGGAGGTTGTAGCGAGCCe-49
```
